# Insecticide resistance in *Anopheles stephensi* in Somali Region, eastern Ethiopia

**DOI:** 10.1186/s12936-020-03252-2

**Published:** 2020-05-12

**Authors:** Solomon Yared, Araya Gebressielasie, Lambodhar Damodaran, Victoria Bonnell, Karen Lopez, Daniel Janies, Tamar E. Carter

**Affiliations:** 1 Department of Biology, Jigjiga University, Jigjiga, Ethiopia; 2 Department of Zoological Sciences, Addis Ababa University, Addis Ababa, Ethiopia; 3 Institute of Bioinformatics, University of Georgia, Athens, GA USA; 4 Department of Molecular Biology and Biochemistry, Pennsylvania State University, State College, PA USA; 5 Department of Bioinformatics and Genomics, University of North Carolina at Charlotte, Charlotte, NC USA; 6 Department of Biology, Baylor University, Waco, TX USA

**Keywords:** Malaria, *Anopheles stephensi*, Insecticide resistance, Kebri Dehar, Somali Region

## Abstract

**Background:**

The movement of malaria vectors into new areas is a growing concern in the efforts to control malaria. The recent report of *Anopheles stephensi* in eastern Ethiopia has raised the necessity to understand the insecticide resistance status of the vector in the region to better inform vector-based interventions. The aim of this study was to evaluate insecticide resistance in *An. stephensi* in eastern Ethiopia using two approaches: (1) World Health Organization (WHO) bioassay tests in *An. stephensi;* and (2) genetic analysis of insecticide resistance genes in *An. stephensi* in eastern Ethiopia.

**Methods:**

Mosquito larvae and pupae were collected from Kebri Dehar. Insecticide susceptibility of *An. stephensi* was tested with malathion 5%, bendiocarb 0.1%, propoxur 0.1%, deltamethrin 0.05%, permethrin 0.75%, pirimiphos-methyl 0.25% and DDT 4%, according to WHO standard protocols. In this study, the knockdown resistance locus (*kdr*) in the voltage gated sodium channel (*vgsc*) and *ace1R* locus in the acetylcholinesterase gene (*ace*-*1*) were analysed in *An. stephensi*.

**Results:**

All *An. stephensi* samples were resistant to carbamates, with mortality rates of 23% and 21% for bendiocarb and propoxur, respectively. Adult *An. stephensi* was also resistant to pyrethroid insecticides with mortality rates 67% for deltamethrin and 53% for permethrin. Resistance to DDT and malathion was detected in *An. stephensi* with mortality rates of 32% as well as *An. stephensi* was resistance to pirimiphos-methyl with mortality rates 14%. Analysis of the insecticide resistance loci revealed the absence of *kdr* L1014F and L1014S mutations and the *ace1R* G119S mutation.

**Conclusion:**

Overall, these findings support that *An. stephensi* is resistant to several classes of insecticides, most notably pyrethroids. However, the absence of the *kdr* L1014 gene may suggest non-target site resistance mechanisms. Continuous insecticide resistance monitoring should be carried out in the region to confirm the documented resistance and exploring mechanisms conferring resistance in *An. stephensi* in Ethiopia.

## Background

Malaria remains a major health problem in 2017, an estimated 219 million cases of malaria occurred worldwide, with 435,000 deaths [1]. In Ethiopia, malaria remains a major public health concern with millions of cases and thousands of deaths reported annually [2]. Unlike most of the African continent, malaria can be caused by infection with *Plasmodium vivax* or *Plasmodium falciparum*. Efforts to control the transmission of malaria currently target *Anopheles arabiensis*, the primary malaria vector in Ethiopia, as well the secondary vectors *Anopheles funestus*, *Anopheles pharoensis*, and *Anopheles nili* [3]. Successes in reducing the malaria burden could be threatened by the recent detection of the South Asian urban vector *Anopheles stephensi* in the Horn of Africa, as its role in malaria transmission in Ethiopia is not yet confirmed. This mosquito was first detected in the Somali Regional State of Ethiopia in 2016 [4] and has subsequently been confirmed to have a broad distribution in Northeast and east Ethiopia [5]. *Anopheles stephensi* was also been reported in Djibouti in 2014 [6] and there are now concerns that this species may spread throughout the African continent [7].

In the past decade, Ethiopia has made significant progresses in expanding coverage of key malaria interventions throughout the country. Indoor residual spraying (IRS) and long-lasting insecticidal nets (LLINs) are used in malaria prevention and control strategy in Ethiopia [8]. IRS was first introduced in Ethiopia in 1959 and continues to use as main malaria intervention method. LLINs distributed throughout Ethiopia particularly in Somali Region, about 80 percent of existing LLINs in households were used the night before and the proportion of LLINs used in malarious areas [9]. In Somali region carbamate insecticides have been frequently sprayed during active malaria season in collaboration by Federal Ministry of Health (FMOH) with the regional Health Bureau.

One major obstacle to vector control in Ethiopia and elsewhere is the ever-developing insecticide resistance as a result of indiscriminate and rampant use of the synthetic chemicals in public health and agriculture [10–12]. Pyrethroids remain the only class of insecticides recommended for the treatment of LLINs and accounted for a large proportion of the insecticide used for IRS in Ethiopia and elsewhere in Africa [8, 13]. This heavy reliance on a single insecticide class has caused mosquito species to develop insecticide resistance. In mosquitoes, pyrethroid resistance is mainly attributed to two major mechanisms: target-site insensitivity and metabolic-based resistance. Target-site resistance is due to mutations in the voltage-gated sodium channel on the mosquito’s neurons that prevent the insecticide’s ability to interfere with the closing of sodium channel that would usually result in paralysis (knockdown) [14]. The knockdown (*kdr*) mutation L1014 has been observed across multiple Culicidae. Metabolic resistance mediated by detoxifying enzymes also plays a significant role in insecticide resistance in malaria vectors [15, 16]. The over-expression of detoxification enzymes such as cytochrome P450 monooxygenases (P450s), carboxylcholinesterases (CCEs) and glutathione S-transferases (GSTs) in mosquitoes are frequently associated with resistance to different classes of insecticides [17].

Insecticide resistance in *An. stephensi* has been reported in Afghanistan, Pakistan, Dubai, and India [18–21]. In these regions, the frequency of the *kdr* L1014 mutation varies with strong support for metabolic resistance as well as target site resistance playing a role in *An. stephensi*. In Ethiopia, studies on *An. arabiensis* in the western portion of the country report phenotypic resistance to pyrethroids along with L1014 variant [22]. In addition, in Southwest Ethiopia, pre-exposure of *An. arabiensis* to piperonyl butoxide (PBO) significantly increased vector susceptibility to deltamethrin and permethrin, suggesting both metabolic and target-site mutation mechanisms are responsible for insecticide resistance [23]. Data on the insecticide resistance status of malaria vectors in the eastern portion of the country is lacking, including that of the recently identified *An. stephensi*. Knowing the status of insecticide resistance of local malaria vectors can aid with vector control planning that involves the use of insecticides. Here the aim of this study was to determine the insecticide susceptibility status of east Ethiopian *An. stephensi* using bioassay tests and characterizing resistance mechanisms using molecular analysis.

## Methods

### Study area

Our samples were collected in Kebri Dehar, a small town in the Somali Regional State, as previously detailed [4]. This town has a tropical semiarid climate with typically bimodal rainfall patterns. The area is also known to experience recurrent droughts. The population size is over 100,000 individuals, many of whom are pastoralists.

### Larval sampling and rearing procedure

**Fig. 1:**
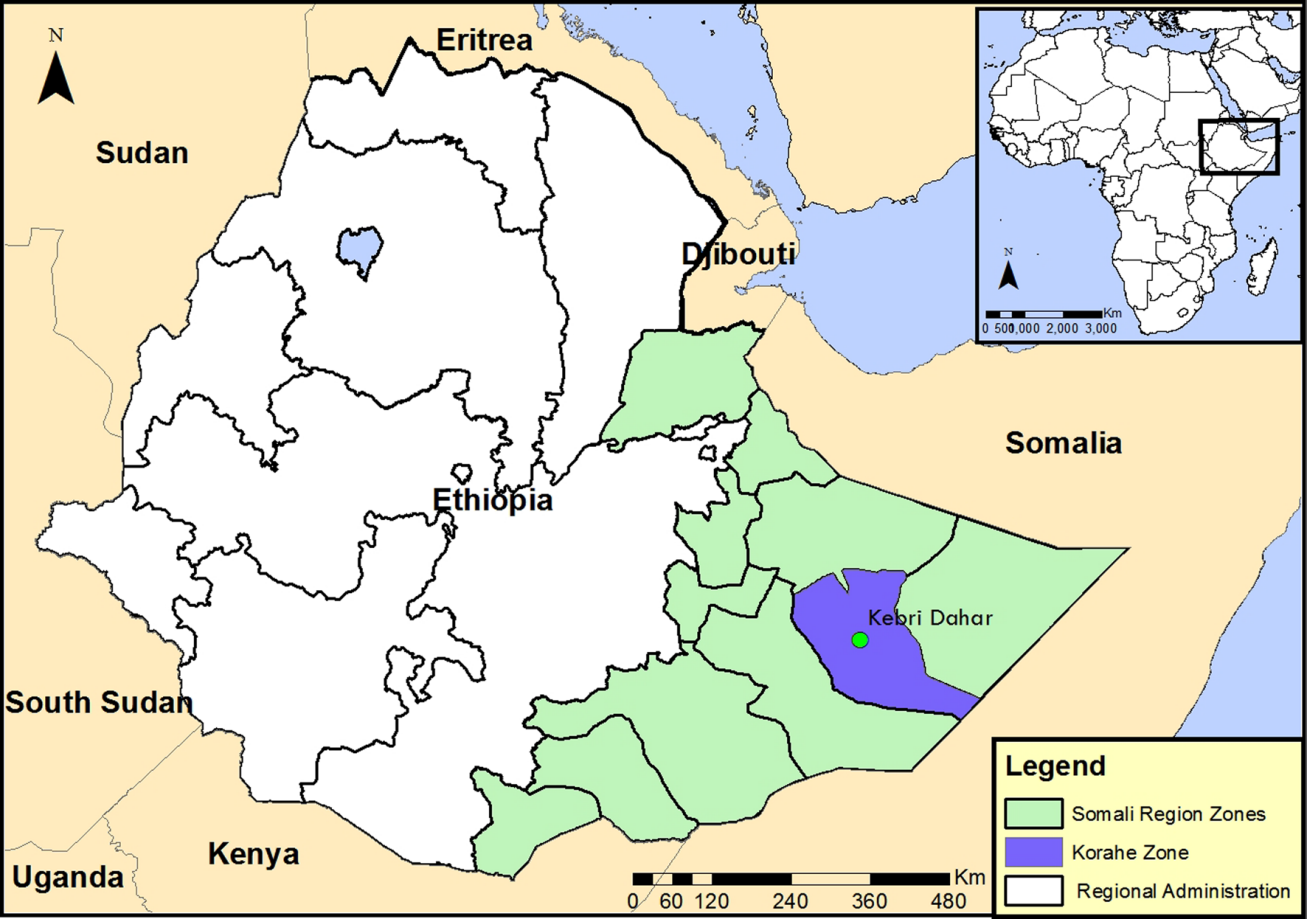
Study map

**Fig. 2:**
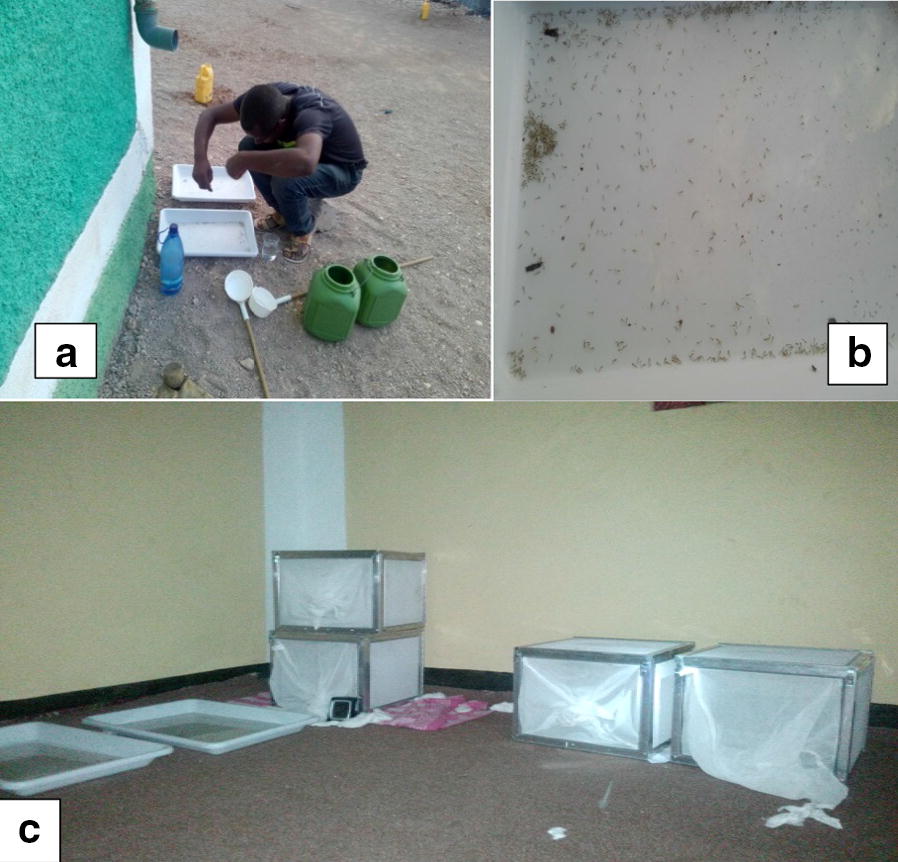
Rearing larvae to adult at field laboratory: **a** Feeding yeast to larvae, **b** larvae and pupae, **c** emerging adult in side cages

Larvae samples were collected from Kebri Dehar small town in Somali Region in eastern Ethiopia as previously detailed (Fig. 1) [4]. This region is predominantly lowland plain, with sparse vegetation including trees and shrubby. Larvae and pupae of *Anopheles* were collected from likely larval breeding habitats including man-made water containers, fresh water pools, stream margins, discarded tires, plastic containers, cisterns, barrels. In the study area water is stored in a container locally called “Birka” and it is constructed from cement and stone, the local people used for drinking and construction purposes. Briefly, immature stages of *An. stephensi* were collected from breeding sites in Kebri Dehar town using the dipping method according to WHO guidelines and were reared to adulthood in the field laboratory (Fig. 2). Collections took place from November to December 2016. In the field laboratory, the larvae and pupae were maintained at 28 ± 2 °C and 70 ± 10% relative humidity. The pupae were sorted and transferred with pipettes from the enamel trays to beakers with small amounts of water. Each beaker was placed inside a cage and was provided with 10% sugar solution for rearing them in the cage (Fig. 2). After two to three days, the pupae emerged to adults and the cages were put in safe place protected from contamination, ants, and other insects. The laboratory reared females of *An. stephensi* were used for different insecticide susceptibility test using WHO bioassay.

### Mosquito identification

Species identification of the mosquitoes was conducted using standard morphological keys [24, 25] and molecular analysis of ITS2 and COI loci as reported previously for *An. stephensi* [4].

### Insecticide susceptibility test for *Anopheles stephensi*

Insecticide susceptibility tests were carried out following WHO insecticide susceptibility test procedure [26]. A total of 700 non-blood fed adult female *An. stephensi* (2–3 day-old) were exposed to insecticide impregnated papers with discriminating concentrations of DDT (4%), Pirimiphos-methyl 0.25%, malathion (5%), deltamethrin (0.05%), permethrin 0.75%, bendiocarb (0.1%), and propoxur (0.1%). Batches of 25 mosquitoes in four replicates were exposed to insecticide impregnated papers for 1 h in WHO test tubes. The knockdown effects for all tested insecticides were recorded at 10, 15, 20, 30, 40, 50, and 60 min. A control in two replicates (50 female *An. stephensi* were used for each insecticide), each with equal number of mosquitoes, exposed to papers impregnated with oil was run concurrently. Mosquitoes were then transferred into holding tubes with untreated papers, where they were supplied with 10% sucrose solution. And, mortality was recorded 24-h post exposure. When mosquito mortality rate in the control is between 5 and 20%, mortality was corrected using Abbott’s formula [27]. All survived and dead specimens following bioassay were kept individually in Eppendorf tubes in silica gel for further molecular identification and *kdr* PCR assays. Determination of resistance was based on WHO criteria as follows: 98–100% mortality indicates susceptibility, 90– 97% mortality indicates resistance candidate (more investigation is needed) and less than 90% mortality suggests resistance [26].

### Amplification and sequencing of insecticide resistance loci

In order to evaluate the presence of insecticide resistance mutations in the *An. stephensi* collected, a portion of *An. stephensi* from each experimental arm were selected for genotyping. For kdr mutation analysis, PCR was used to amplify the region of the *vgsc* gene that housed the L1014 alleles. Mosquito legs were used as templates for DNA. Mosquitoes were PCR amplified and sequenced individually. Analysis of the *vgsc* was completed according to Singh et al. [20]. PCR amplification of a portion the acetylcholinesterase gene (*ace*-*1*), associated with resistance to organophosphates and carbamates [28] was also performed for *An. stephens*i according to the protocol detailed in [29]. *Vgsc* and *ace*-*1* PCR reactions were performed at 25 μl total with 2X Promega Hot Start Master Mix (Promega, Madison, W), with 1 μl template DNA, and the primer conditions listed in Table 1. Temperature protocols for *vsgc* amplification were as follows 95 for 5 min, 35 cycles of 95 for 30 s, 50 for 30 s, 72 for 45 s, 72 for 7 min. Temperature protocols for *ace*-*1* were as follows: 94 for 5 min, 35 cycles of 94 for 30 s, 54 for 30 s, and 72 for 30 s, 72 for 5 min. PCR products were run on 2% agarose gel for 1 h at 100 V to confirm successful PCR amplification.

**Table 1:** List of primer and conditions used for PCR amplification of portions of the voltage gated sodium channel and acetylcholinesterase genes

Assay	Primer	Sequence	Annealing temperature (°C)	Final concentration (µM)
Voltage gated sodium channel				
	KdrF	GGACCAYGATTTGCCAAGAT	50	1.25
	VGS_1R	CGAAATTGGACAAAAGCAAGG	50	1.25
Acetylcholinesterase				
	Ex3AGdir	GATCGTGGACACCGTGTTCG	56	1
	Ex3AGrev	AGGATGGCCCGCTGGAACAG	56	1

### Analysis of *vgsc* and *ace*-*1* sequences

Sequences were submitted as queries to the National Center for Biotechnology Information’s (NCBI) Basic Local Alignment Search Tool (BLAST) to confirm correct loci were amplified. Sequences were then aligned to identify *kdr* L1014 and *ace*-*1R* G1109 mutations. The allele and genotype frequencies of these mutations were then calculated.

## Results

### *Anopheles stephensi* insecticide resistance

A total of 1200 *An. stephensi* larvae and pupae were collected from the breeding sites. *Anopheles stephensi* larvae occurred more frequently in cemented water reservoir and plastic water reservoir for construction. *Anopheles stephensi* positive habitats were mainly located close to human dwelling. Other Larvae and pupae of *Aedes* and *Culex* mosquitoes were visually detected and coexisted with *An. stephensi*, but not recorded.

### Bioassay results

A total of 700 *An. stephensi* were tested with different insecticides based on WHO protocol. The results of the susceptibility status of populations of *An. stephensi* are presented in Table 2. Overall, the percent mortality after exposure to insecticides ranged from 14% (pirimiphos-methyl) to 67% (deltamethrin). Using the WHO mortality threshold of above 98%, *An. stephensi* demonstrated resistance to bendiocarb, propoxur, DDT, malathion and permethrin.

**Table 2:** Percentage of mortality of *Anopheles stephensi* in different insecticide in Kebri Dehar town

Insecticides discriminating concentration (%)	Classification	No. of *An. stephensi* tested	Mortality rate after 24 h (%)	Resistance? (< 98%)
Bendiocarb 0.1%	Carbamates	100	23	Yes
Propoxure 0.1%	Carbamates	100	21	Yes
Deltamethrin 0.05%	Pyrethroid	100	67	Yes
Permethrin 0.75%	Pyrethroid	100	53	Yes
Malathion 5%	Organophosphates	100	32	Yes
DDT 4%	Organochlorine	100	32	Yes
Pirimiphos-methyl 0.25%	Organophosphates	100	14	Yes

### *Anopheles stephensi* insecticide resistance mutations

A total of 51 mosquitoes were selected randomly from each research arm to represent the natural population of *An. stephensi*, including 19 that were tested for resistance to deltamethrin, permethrin, or DDT. Of these, eight were resistant to one of these insecticides. Of the 51 *An. stephensi* examined for *kdr* mutations, none carried the L1014 mutation. In addition, 30 *An. stephensi* were analysed for ace1 mutations. Of these, 20 had been tested for resistance to bendiocarb, propoxur, or malathion and eight were found to be resistant. Overall, none of the *An. stephensi* genotyped carried the *ace1R* G119S mutation.

## Discussion

This is the first report of *An. stephensi* in Ethiopia exhibiting insecticide resistance. What is most concerning is that *An. stephensi* showed resistance to seven insecticides included in this study highlighting a potential challenge with insecticide-based vector control in this region. This could be *An. stephensi* is quickly adapting and invading new environment, even survives extremely high temperatures during the dry season [7]. There is some consistency with previous studies on *An. stephensi* resistance to insecticides. As in the present study, *An. stephensi* was shown to be resistant to DDT in Iran [30] (Gorouhi et al. 2016). Similarly, a study on *An.* *stephensi* in Afghanistan revealed resistance to deltamethrin, malathion, permethrin and DDT [31]. In addition, *An. stephensi* carbamate resistance was observed in a recent study in Iran [32] as observed in the present study. However, there were some difference between our findings on *An. stephensi* and previous reports, where *An. stephensi* was found to be susceptible to the pyrethroids (deltamethrin and permethrin) and malathion in Iran [30, 33] and Pakistan [34]. These differences may reflect differences in the type and extent of insecticide use in Ethiopia compared to other countries.

One surprising finding from our study was the absence of *kdr* mutation with phenotypic evidence of pyrethroid resistance. The absence of *kdr* mutations in pyrethroid resistant *Anopheles* is rare but not unprecedented. A study conducted on *An. stephensi* collected in Afghanistan revealed a low frequency of L1014 wild-type mutation (44%) in mosquitoes and a lack of homozygotes of the mosquitoes that were resistant to deltamethrin [35]. The phenotypic presentation of resistance in the majority of *An. stephensi* specimens with the absence or low frequency of *kdr* mutations may suggest that metabolic resistance as opposed to targeted resistance is the primary resistance mechanisms in the Ethiopian *An. stephensi*. A follow-up study on cytochrome P450s, esterases, glutathione S-transferases (GSTs) and acetylcholine esterase (AChE) activities in pyrethroid resistant mosquitoes in Afghanistan further highlight the role of metabolic resistance in *An. stephensi* [21]. It is also possible the other variants in the *vgsc* gene may lead to resistance. Additional sequencing and analysis of the entire *vgsc* should be conducted for identification of other mutations that could lead insecticide resistance.

There are some similarities between these findings on *An. stephensi* resistance and what has been reported in *An. arabiensis* in Ethiopia. In *An. arabiensis*, resistance has been reported for insecticides belonging to all four chemical classes approved for IRS and LLINs. These include DDT (organochlorine), malathion (organophosphate), bendiocarb and propoxur (carbamates) and alpha-cypermethrin, cyfluthrin, deltamethrin, etofenprox, lambda-cyhalothrin and permethrin (pyrethroids) [36–40]. However, the frequencies of *kdr* mutations are much higher in the Ethiopian *An. arabiensis* [36, 40] than what is reported here. These findings suggest that while, insecticides may induce the development of resistance over time, mechanisms for resistance may vary across species. In depth cross-species genetic analysis for selective signatures on the *vgsc* and unidentified loci across the genome are needed and underway to further elucidate the differing mechanisms for insecticide resistance in *Anopheles* species.

In the present study, no *ace*-*1* mutations were observed. There is still some ambiguity around the significance of the *ace*-*1* mutation has been proposed to induce resistance to organophosphates and carbamates resistance [28]. The mutation was absent in the *An. stephensi* tested in the present study this may reflect the history of the type of insecticides used in the region.

## Conclusion

The finding of multi-insecticide resistance in *An. stephensi* in Kebri Dehar, Ethiopia emphasizes the need for additional investigation in other parts of Ethiopia and the Horn of Africa. These findings are of importance in the planning and implementation of malaria vector control strategy in the region. Additionally, only a portion of the available insecticides were tested in this study. Resistance investigations should include some of the other compound classes including lambda-cyhalorthrin, temephos and chlorpyriphos. The complete absence of the mutation is unexpected but may be due to the *An. stephensi* collection being limited to one site. Future studies should evaluate the frequency of this mutation in other regions of Ethiopia. The Federal Ministry of Health of Ethiopia should implement appropriate resistance management strategies and integrated vector control intervention.

## Data Availability

The datasets analysed in this study are available from the corresponding author on reasonable request.

## Abbreviations

ace-1: acetylcholinesterase gene
BLAST: Basic Local Alignment Search Tool
cox1: cytochrome c oxidase subunit 1
CCEs: carboxylcholinesterases
DNA: deoxyribonucleic acid
FMOH: Federal Minster of Health
GSTs: glutathione S-transferases
ITS2: internal transcribed spacer 2
IRS: Indoor Residual Spraying
LLINs: Long Lasting Insecticidal Nets
kdr: knockdown resistance
NCBI: National Center for Biotechnology Information
PCR: polymerase chain reaction
RNA: ribonucleic acid
SRS: Somali Regional Sate
vgsc: voltage gated sodium channel
WHO: World Health Organization
